# Measurement of Magnetic and Magnetostrictive Characteristics of Transformer Core Based on Triaxial Strain Gauge and *B*-*H* Vector Sensor

**DOI:** 10.3390/s23135926

**Published:** 2023-06-26

**Authors:** Zhen Wang, Zheming Fan, Xiang Li, Kai Xu, Runjie Yu

**Affiliations:** 1 School of Electrical Engineering, Shenyang University of Technology, Shenyang 110870, China; 2 Shenyang Railway Prospecting Design Institute Co., Ltd., Building Electrification Institute, Shenyang 110000, China

**Keywords:** strain gauge, ***B***-***H*** vector sensor, transformer core, magnetostriction, lamination

## Abstract

As is well known, the magnetostrictive phenomenon of electrical steel sheet is the main source of electricity in equipment such as transformers. The magnetostrictive characteristic of the actual transformer core is more complicated than that of single-sheet steel. The magnetostriction phenomenon of the transformer core cannot be fully understood by studying only a single piece of electrical steel, so it is necessary to study the local magnetic characteristics of the transformer directly. In this paper, two-limb, one-phase transformer core with a multi-step-lap construction was assembled, a laminated magnetostrictive testing system based on triaxial strain gauges was built, and the local magnetic characteristics were studied using a self-developed ***B***-***H*** vector sensor. The magnetostrictive and magnetic properties in different local regions were measured and analyzed under several magnetization patterns, and the influence of DC bias on the magnetostrictive property of the corner, yoke, and limb of the core was investigated. The influence of the position of the clamp on the magnetostriction of the transformer core was also studied. The magnetostrictive strain of the single sheet and laminated core was compared and discussed. The results showed that the strain caused by the interaction between laminations in this area can be effectively reduced when clamping in the middle of the yoke.

## 1. Introduction

Recently, the study on the vibration of the transformer has drawn more and more attention due to growing environmental awareness [1,2]. Severe vibration will cause various damages to equipment. The low-frequency vibration noise of electrical equipment such as motors and transformers will not only interfere with the lives of residents, but will also have an impact on their mental health. In high-voltage direct-current (HVDC) systems, unexpected bias current will appear in the transformer, which will lead to a series of electromagnetic effects caused by the DC magnetic potential or DC magnetic flux in the magnetic core, making the transformer work in an abnormal state [3,4].

Scholars have conducted extensive research on the vibration and noise of motors and transformers. Peng et al. applied an optimization algorithm combined with the 3D FEM and Taguchi optimization method to the tooth shape of the three-phase hybrid stepper motor to reduce the noise caused by harmonics in the “torque-angle characteristics” of the motor [5]. Hu et al. analyzed the critical vibration suppression of the maglev high-speed motor with μ-synthesis control [6]. Suawa et al. used vibration and sound data sets from sensors to study the example of coordinating the optimal combination of sensors with a deep learning sensor fusion algorithm, to achieve predictive maintenance of the BLDC motor [7]. In order to reduce noise in the brushless direct-current motor and obtain a reliable optimization geometry of noiseless seat motion, Lee et al. conducted parametric analysis of a brushless DC motor through the design of experiments and Monte Carlo statistical analysis, and determined the optimal slot depth and stator tooth width by using a non-linear prediction model [8]. Martinez et al. detected the occurrence of iron resonance events by utilizing noise caused by the magnetostrictive force in the core of the inductor voltage transformer [9]. Bichurin et al. introduced multiple ME magnetic field sensors. Through the analysis of the characteristics of the sensors, they showed that they have broad application prospects in engineering [10]. Monteiro et al. suggested that optical fiber sensors be used to monitor the structure of power transformers and realize real-time monitoring of the structure of power transformers [11]. Based on statistical time characteristics (STFs) and support vector machines (SVMs), Huerta-Rosales et al. employed the vibration signals produced in the transformer to diagnose winding faults [12]. Grenda et al. developed a new magnetomechanical force sensor with higher sensitivity based on the characteristics of transformer steel [13]. Kim et al. proposed a method to classify the over-, normal-, and under-voltage levels supplied to the transformer using the acoustic signal of the transformer operating in various noise environments [14]. Li et al. established an engineering model of the magnetic flux density and electromagnetic force density on transformer core discontinuities, and then analyzed the important sources of transformer vibration and noise [15]. By comparing the acoustical parameters of the core model with the 500 kV transformer, Zhou et al. selected three typical parameters for DC bias state acoustic warning, and clear parameter warning thresholds were given [16]. He et al. proposed a method to evaluate the effectiveness of transformer online monitoring data under DC bias driven by monitoring data under different working conditions [17].

The DC magnetic bias phenomenon is a kind of abnormal working condition of the power transformer, which will cause a DC component in the transformer winding and then lead to DC flux in the core. A series of electromagnetic effects can also occur. Under DC bias, the core magnetostriction effect is more intense, the vibration is intensified, the noise level is increased, and even the transformer body can become permanently damaged in serious cases. The concept of the magnetic domain is introduced to explain the magnetostriction phenomenon from the mesoscopic scale. The ferromagnet will be accompanied by the movement of the magnetic domain wall and the rotation of the magnetic moment of the magnetic domain in the magnetization process, and magnetostriction mainly occurs in the rotation of the magnetic moment of the magnetic domain. On the one hand, some methods about reducing the noise have been developed by means of adjusting the overlapping method and clamping pressure on the cores [18,19,20,21]. On the other hand, magnetostrictive properties are being studied by carrying out some experimental tests on a single steel sheet under an alternating magnetization [22,23]. However, it will be more significant to investigate the magnetostrictive strain in the laminated core because the magnetostrictive properties in the transformer core will be different from those in a single steel sheet due to its complicated lamination configuration and magnetization patterns in the engineering application.

In this paper, an experimental setup was built to study the magnetic and magnetostrictive strain signals on the local regions of a single-phase transformer core configuration. The localized magnetic and magnetostrictive characteristics under different magnetization patterns are presented and the effects of the DC-biased magnetic field on the property above are discussed. The influence of the position of the clamp on the magnetostriction of the core was also studied. This work may be helpful for a deeper study of transformer vibration and noise.

## 2. Magnetostriction in Transformer Core

**Figure 1:**
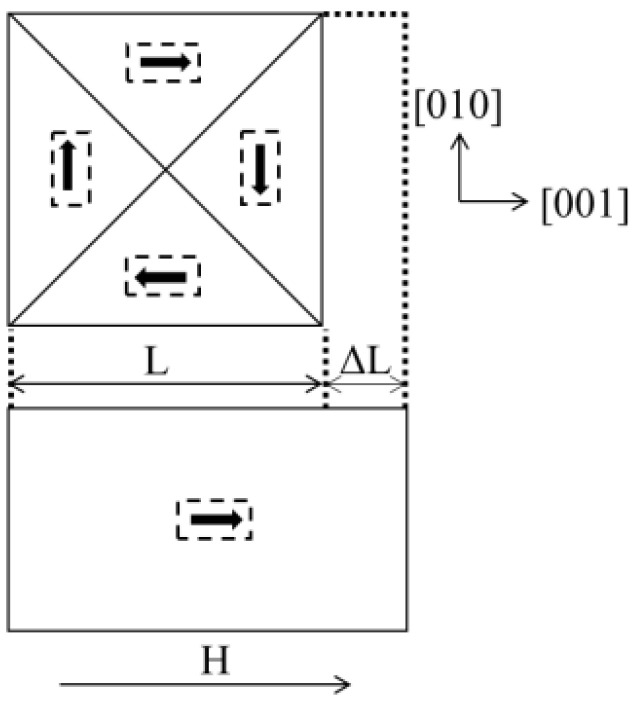
Micromechanics of magnetostriction.

Accurate measurement of the magnetostrictive characteristics of the electrical steel sheet is the fundamental guarantee of magnetostrictive characteristics research, and the premise and basis of effective modeling and simulation. Magnetostriction refers to the change in the shape and size of a material in the magnetized state under the action of an external magnetic field. Magnetostriction can be explained from a microscopic perspective by introducing the concept of magnetic domains, which are small areas of neatly aligned spin magnetic moments and saturated magnetization. In the magnetization process of a ferromagnet, there is no magnetic field in the first stage, the magnetic domain is disordered, and the magnetic moment of the ferromagnet is 0. During the magnetization of the ferromagnet in an external magnetic field, there is no magnetic field in the first stage, the magnetic domain is disordered, and the magnetic moment is 0. In the second stage, a magnetic field is added. With the increase in the external magnetic field, the domain displacement and rotation between magnetic domains occur, and the domain orientation tends to be consistent. The third stage is a stable state, where the domain orientation is basically the same and the total energy is lowest. Macroscopically, the magnetostriction phenomenon can be observed, as shown in Figure 1.

Magnetostriction is independent of the symbol of magnetic flux, and the unit of the magnetostrictive shape variable is μm/m or nm/m. This results in the fundamental frequency of magnetostriction being twice the frequency of the excitation signal and the harmonics being integral multiples of the excitation signal. The influence of magnetostrictive harmonics becomes more important because of the response characteristics of the human ear.

### 2.1. Experimental Setup and Approach

**Figure 2:**
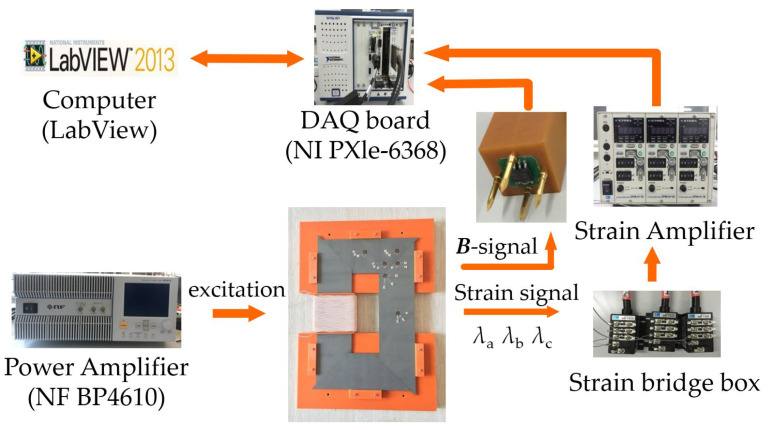
Experimental setup of a single-phase transformer core.

**Figure 3:**
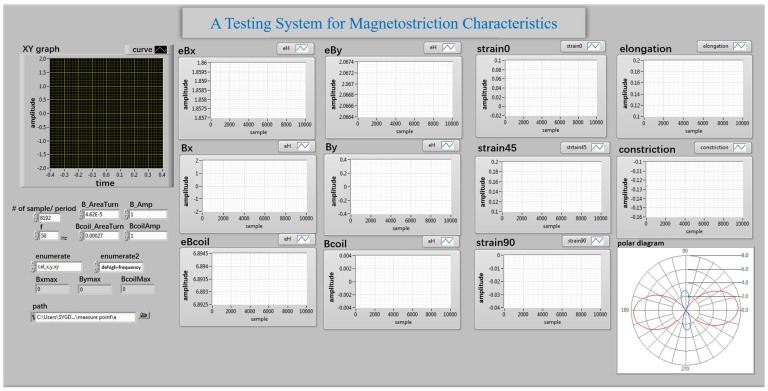
The program interface of magnetostrictive characteristic testing.

An overview of a single-phase transformer core testing system is illustrated in Figure 2, which shows the experimental setup of localized magnetic characteristics and magnetostrictive strain measurement, in which a power amplifier is used to supply the windings of the transformer core with an alternating current (ac) or an ac with DC common excitation. The localized magnetic signals are measured by a self-developed ***B***-***H*** vector sensor. The magnetostrictive strain is measured from the resistance strain gauge, KFG-5-120-D17-11, which is attached to the surface of the core to obtain in-plane magnetostriction in any direction; then, they are transferred to a LabVIEW program through a data acquisition board to be processed. The program interface of magnetostrictive testing is illustrated in Figure 3.

In order to measure the magnetostrictive strain of an electrical steel plate along any direction, the triaxial resistance strain gauge method is adopted in this paper. When the sample is magnetized, a small geometric deformation will be generated, which will also be caused by the connected strain gauge. The strain gauge will convert the deformation signal into a resistance change and then convert it into a voltage signal through the strain bridge box. After being amplified by the strain amplifier, the signal will be transmitted to the PC and then converted into the corresponding strain signal. The three directions of the strain gauge are at angles of 0°, 45°, and 90° to the rolling direction of the sample to be measured; the model of the strain gauge sensitivity coefficient is 2.1 ± 1.0%; each axis strain resistance is 120.4 ± 0.4 Ω; the grid length is 10 mm; and the width is 3 mm. The strain gauge is connected to the strain bridge box and strain amplifier, and the three strain signals are passed into the LabVIEW control program of the computer through the BNC adapter and the data acquisition card, to realize the measurement and processing of the signals. Among them, the strain bridge box is the DB-120A series manufactured by KYOWA, and the balance of bridge arm resistance is 120. Since the order of magnitude of the strain signal is small, usually nm/m, the collected signal needs to be amplified by a strain amplifier. In this paper, the DPM-911B series strain amplifier produced by KYOWA Company is selected, which has the function of filtering and amplification at the same time. Device parameters of the magnetostrictive testing system are shown in Table 1.

### 2.2. Measurement Principle of Strain Gauge

Strain gauges, as commonly used in mechanical sensors, can convert small dimensional changes into a resistance change that can then be further converted into a voltage signal. With strain gauges, we can observe deformation around a specific point on the specimen being tested and measure the strength and safety of the sample. Strain gauge measurement devices are often used to detect parameters such as pressure, acceleration, displacement, and torque and are applied in various fields such as mechanical engineering, automotive, electrical engineering, and civil engineering. Resistance strain gauges are one of the most widely used techniques for measuring displacement.

When the sample under test is subjected to an external tensile or compressive force, it is stretched or shortened, and its resistance value increases or decreases accordingly. When the sample under test is subjected to strain ε, it is assumed that the resistance *R* changes Δ*R* under its influence.

(1) $$\frac{\Delta R}{R}=K_{s}\cdot \varepsilon$$

where strain rate *K*_s_ represents the coefficient of sensitivity.

**Figure 4:**
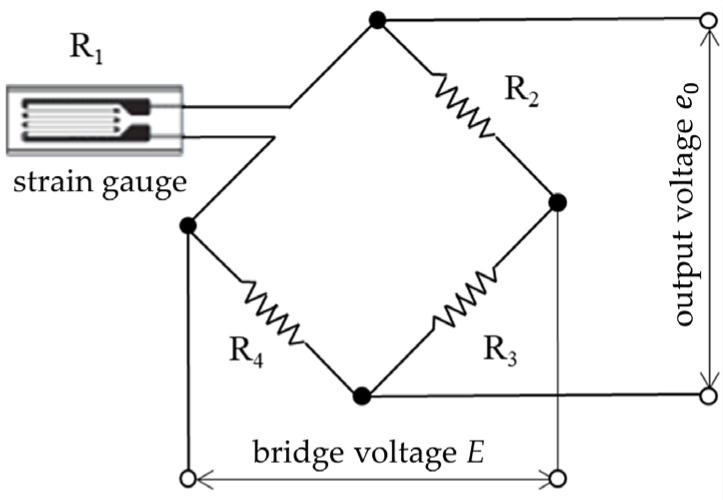
Wheatstone bridge.

When the strain gauge is used alone, considering that the resistance variation caused by the strain gauge is very small, voltage detection needs to be completed by a Wheatstone bridge, as shown in Figure 4. The resistances on the bridge are *R*_1_, *R*_2_, *R*_3_, and *R*_4_ (Ω). When the bridge voltage is *E* (V), the output voltage *e*_0_ (V) is

(2) $$e_{0}=\frac{R_{1}R_{3}-R_{2}R_{4}}{\left(R_{1}+R_{2})(R_{3}+R_{4}\right)}\cdot E$$

When resistance *R*_1_ is the strain gauge, if *R*_1_ only changes Δ*R*, then

(3) $$e_{0}=\frac{(R_{1}+\Delta R)R_{3}-R_{2}R_{4}}{\left(R_{1}+\Delta R+R_{2})(R_{3}+R_{4}\right)}\cdot E$$

If *R*_1_ = *R*_2_ = *R*_3_ = *R*_4_ = *R*, then

(4) $$\begin{array}{ll} e_{0} & =\frac{R^{2}+R\Delta R-R^{2}}{(2R+\Delta R)2R}\cdot E\\ & =\frac{\Delta R}{4R+2\Delta R}\cdot E \end{array}$$

Because *R* is much bigger than delta Δ*R*,

(5) $$e_{0}\approx \frac{1}{4}\cdot \frac{\Delta R}{R}\cdot E=\frac{1}{4}\cdot Ks\cdot \varepsilon \cdot E$$

By connecting the output voltage *e*_0_ to the strain amplifier, the signal can be further amplified and the output voltage proportional to the strain gauge can be determined.

The measurement accuracy of the strain gauge is closely related to the adhesive of the strain gauge. The adhesive suitable for the characteristics of the material to be measured and in line with the working temperature should be selected and pasted on the surface of the component to be measured. During the measurement, the local deformation of the component to be measured will be transmitted to the base of the strain gauge sensor through the adhesive, to realize local deformation detection.

The magnetostrictive strain in any direction in the plane can be calculated by the following formula [24]:

(6) $$\lambda \left(t,\theta \right)=\varepsilon_{x}\left(t\right)\cos^{2}\theta +\gamma_{\mathrm{xy}}\left(t\right)\sin\theta \cos\theta +\varepsilon_{y}\left(t\right)\sin^{2}\theta$$

where *ε*_x_ and *ε*_y_ are the linear strains and *γ*_xy_ is shear strain that can be achieved by

(7) $$\left[\begin{array}{c} \varepsilon_{x}\left(t\right)\\ \varepsilon_{y}\left(t\right)\\ \gamma_{\mathrm{xy}}\left(t\right) \end{array}\right]={\left[\begin{array}{ccc} \cos^{2}\theta_{a} & \sin^{2}\theta_{a} & \sin\theta_{a}\cos\theta_{a}\\ \cos^{2}\theta_{b} & \sin^{2}\theta_{b} & \sin\theta_{b}\cos\theta_{b}\\ \cos^{2}\theta_{c} & \sin^{2}\theta_{c} & \sin\theta_{c}\cos\theta_{c} \end{array}\right]}^{-1}\cdot \left[\begin{array}{c} \lambda_{a}\left(t\right)\\ \lambda_{b}\left(t\right)\\ \lambda_{c}\left(t\right) \end{array}\right]$$

where the magnetostrictive strain *λ*_a_, *λ*_b_, and *λ*_c_ is obtained from the three directions of the resistance strain gauge.

The principal strain of elongation and contraction *λ*_p_^+^(t) and *λ*_p_^−^(t), respectively, can be expressed as

(8) $$\lambda_{p}^{+}(t),\lambda_{p}^{-}(t)=\frac{\varepsilon_{x}(t)+\varepsilon_{y}(t)}{2}\pm \sqrt{{\left[\frac{\varepsilon_{x}(t)-\varepsilon_{y}(t)}{2}\right]}^{2}+{\left[\frac{\gamma_{\mathrm{xy}}(t)}{2}\right]}^{2}}$$

**Figure 5:**
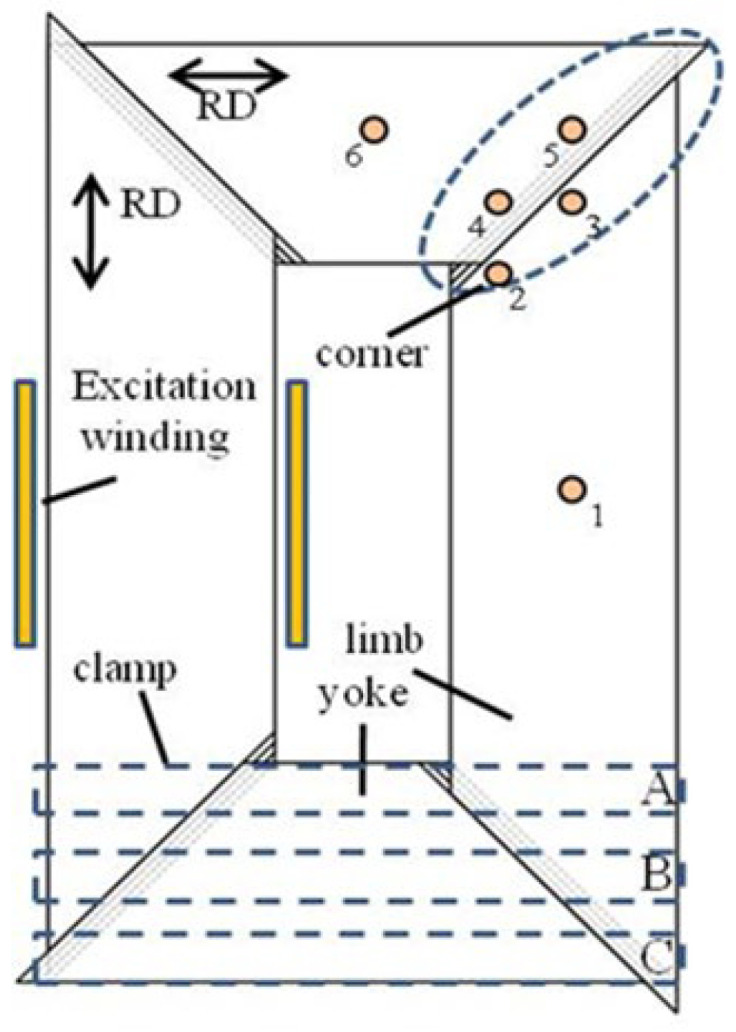
Schematic diagram of laminated core.

The 2-limb, 1-phase transformer core is stacked from 12 layers of grain-oriented electrical steel sheets. The core size is 385 mm × 250 mm and the widths of both the yoke and limb are 90 mm. A multistep-lap construction of four steps is employed. The six measurement points “1–6” of interest are distributed on the yoke, limb, and corner of core, as shown in Figure 5.

### 2.3. The Effect of Clamping Position on Magnetostrictive Characterizations of Transformer Core

The influence factors of transformer core vibration include not only those related to the overlapping form of the core, but also the clamping position. In this investigation, 250 mm × 20 mm wooden plates are positioned, marked with “A/B/C” on both the upper yoke and lower yoke, as shown in Figure 5. To focus mainly on the effect of clamping position on the magnetostrictive strain of the core, there are no bolts to be used to fix the core in case the drilling process changes the magnetostrictive strain in the core. The core is clamped by imposing a pressure of 0.5 kN on the wooden plates.

Table 2 lists the measured results of the peak-to-peak value of contractive principal strain *λ*_−pp_ under different clamping positions at six test points when the core is magnetized at 1.5 T. For a fixed clamping position, for example, position “A”, when a clamping pressure of 0.5 kN is applied, the strain located at “*point 3–5*” is obviously larger than those at “*point 1*” and “*point 6*”. The strain at the corner area is almost 1.8 times those on the limb and yoke. This implies that the core strain at the corner is induced not only by the magnetostriction but also the interlaminar dynamic forces between free components of laminations, while the strain of the limb is mainly caused by magnetostriction.

From Table 2, the change in the position of the clamp has less influence on the magnetostrictive strain of the yoke and the limb, but it causes a relatively obvious variation in strain on the corner region. When the clamp is located at position “B”, the strain at “*point 3–5*” is less than that at position “C”. The strain value in the area where the clamp is located will be reduced since the magnetic flux distributes more uniformly there. As a result, the magnetostriction as well as the attractive forces of the lamination area reduce. Therefore, we can conclude that when the clamps are fixed in the middle of the yoke, the overall strain in the corner region decreases. The following research is carried out by means of tightening the wooden plates at clamping position “B” with 0.5 kN is applied.

### 2.4. Magnetostrictive Characterizations in the Transformer Core under Alternating Magnetization

**Figure 6:**
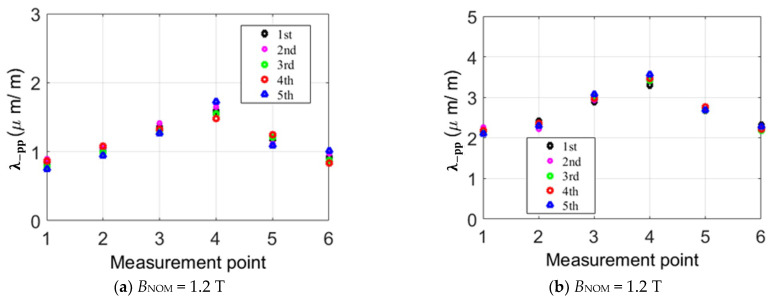
Distribution of *λ*_−pp_ magnetostriction strain at points of interest without a DC bias by repeating measurement processes five times.

In order to minimize measurement uncertainty, the magnetostrictive strain signals at each point of interest on the cores in Figure 5 are repeated five times, and Figure 6 shows the measurement results of principal strain *λ*_−pp_ repeated 5 times when the nominal inductions of *B*_NOM_ = 1.2 T and *B*_NOM_ = 1.7 T are applied. The average values of *λ*_−pp_ at six measurement points at *B*_NOM_ = 1.7 T are 2.19 μm/m, 2.37 μm/m, 2.98 μm/m, 3.46 μm/m, 2.77 μm/m, and 2.22 μm/m, and the maximum measurement deviation is about ±0.30 μm/m. From Figure 6, we observe that the strain values in each region increase with the increase in magnetization strength. Moreover, the strain at “*point 3–5*” is larger than those at the limb and the yoke due to the rotational magnetic flux density occurring at the corner of core, which induces more strain. The existence of a rotating magnetic field is explained in Section 3. Another reason is that the attractive forces are caused by the inter-laminar off-plane flux close to air gaps.

### 2.5. Magnetostrictive Characterizations in the Transformer Core in the Presence of DC Bias

**Figure 7:**
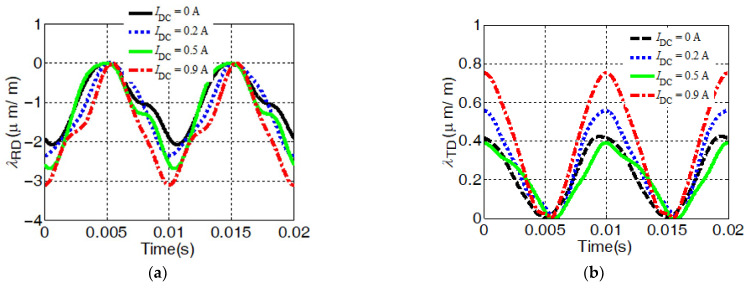

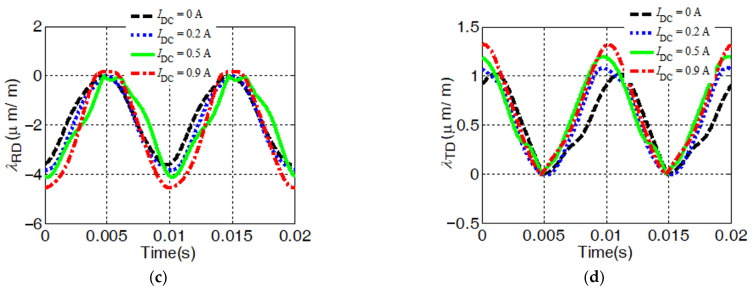
The influence of DC bias on local magnetostriction of core: (**a**) *λ*_RD_ at *point 1*; (**b**) *λ*_TD_ at *point 1*; (**c**) *λ*_RD_ at *point 4*; (**d**) *λ*_TD_ at *point 4*.

Figure 7 discusses the influence of DC bias on the magnetostrictive strain of the core. In this paper, the direct current *I*_DC_ is applied along the same side as the sinusoidal excitation source *I*_ac_, which may reflect the fact of DC bias caused by the monopole earth return of GIC or HVDV. The magnetizing current in the winding is imposed with a DC component of 0.2 A, 0.5 A, and 0.9 A, respectively.

Figure 7a–d indicate measured strain waveforms in one magnetization cycle along both the RD, *λ*_RD_, and the TD, *λ*_TD_, with a DC bias magnetic field when the core is magnetized at 1.7 T. The strain of the magnetostriction in the transform core varies periodically twice within a 0.02 s magnetization period. The strain along the RD exhibits contractive strain and the strain along the TD is positive with the property of elongated strain. The contractive strain *λ*_−pp_ is always greater than the elongation strain. 

In addition, the appearance of DC bias in the excitation current leads to the magnetic field entering the saturation stage and the magnetostrictive strain increasing. However, the influence of DC bias on the magnetostrictive properties of the corner and limb area of the core is different. It can be concluded from Figure 7a,c that when a DC component of 0.9 A is imposed, compared with DC bias, the strain value of *λ*_RD_ at “*point 1*” of the limb increases by 50%; nevertheless, “*point 4*” of the corner area increases by 30%. Similar results are also exhibited for the TD. Thus, in corner region, it reveals that the impact of DC bias is weaker.

### 2.6. Comparison of Magnetostrictive Strain between Single Sheet and Laminated Core

Table 3 lists the comparison of peak-to-peak values of principal strain between the single sheet and column region of the laminated core. Magnetostriction results of the single sheet are measured by the equipment in Reference [25]. It shows that the magnetostrictive strain under the lamination increases slightly compared with that of the single sheet. Especially, with the increase in magnetization intensity and DC bias, the strain amplitude under the lamination will increase, and the maximum amplitude will reach 42%. The magnetostrictive deformation of a single sheet is the magnetostrictive strain caused by standard sinusoidal waveform excitation, while the local magnetic characteristics of the laminated core are complex. Also, the actual magnetization type is not a standard sinusoidal waveform, and the deformation at this time includes not only the magnetostrictive strain but also the deformation caused by the electromagnetic force of the leakage magnetic field.

## 3. Magnetic Characteristics in Transformer Core

In order to better study the causes of the magnetostrictive increase in the core corner area in Section 2, we carry out the experimental test of core magnetic characteristics on six points of interest on the surface of a transformer core by means of a self-developed ***B***-***H*** vector sensor [26]. The ***B***-***H*** sensor can provide the ***B*** signals and ***H*** signals along two orthogonal directions over one magnetization cycle at these points, and then ***B*** trajectories can be achieved. 

**Figure 8:**
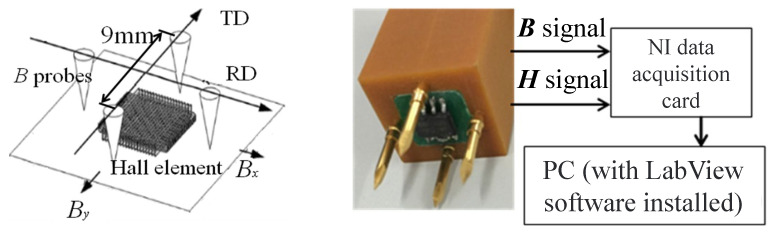
***B***-***H*** vector sensor.

**Figure 9:**
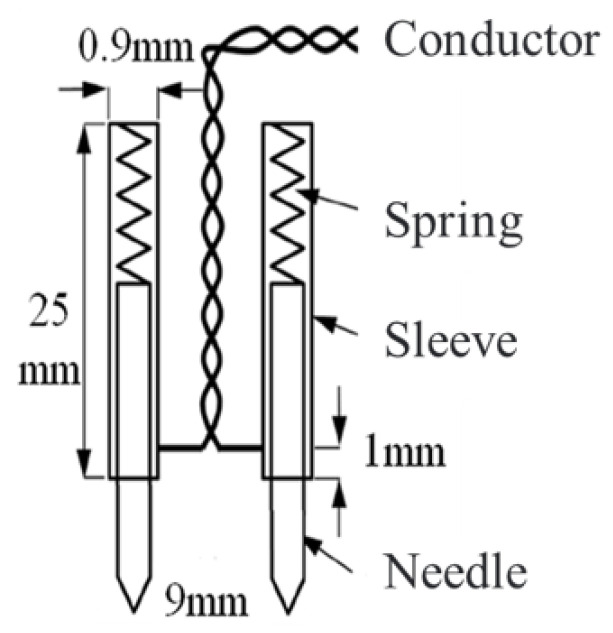
Construction and connection method of the *B* probe.

Figure 8 illustrates the configuration of the ***B***-***H*** vector sensor. The sensor has two sets of ***B*** probes made from non-magnetic material for measuring the induced emf *e*B across the probes. The spacing between the probes is 9 mm and there are hard pins on the end of the probe enough to pierce the insulation coating of the electrical steel sheet to achieve good electrical contact during the measurement. The construction and connection method of the probe are shown in Figure 9, which consists of a sleeve, needle, and spring. The spring supports the needle and is fixed inside the sleeve. During the measurement process, the needle is fully retracted into the sleeve. The needle is made of non-magnetic material and non-magnetic steel, with a relative permeability of 1.0002, which does not affect the measured magnetic field. The probe tip is machined into a triangular cone shape by high-speed rotation of a grinding wheel, which can penetrate the insulation coating of electrical steel sheets and achieve good electrical contact. A wire to the other end of the probe is connected for signal extraction, and the wire is wound into a spiral shape to reduce the impact of stray magnetic flux on the measurement results.

The electromotive force signals induced by magnetic flux changes in two orthogonal directions are measured by two pairs of probes. According to Faraday’s law of electromagnetic induction,

(9) $$e_{Bi}=-N_{Bi}\frac{d\varphi }{dt}=-N_{Bi}\int_{S_{Bi}}\frac{dB_{i}}{dt}ds$$

where $e_{Bi}$ is the measured induced voltage value and subscript *i* = *x* or *y* represents two orthogonal measurement directions; *N_Bi_* is the equivalent number of turns of the probe. Since the probe measures the voltage on one side of the closed loop, which is equivalent to a half-turn coil, *N_Bi_* = 1/2; *S_Bi_* is the effective area of the measuring area of the probe, which is equal to the product of the distance between the relative probes and the thickness of the sample to be measured. *T* is the period. The magnetic flux density value obtained from Equation (9) is

(10) $$B_{i}=\frac{2}{S_{Bi}}\int_{0}^{T}e_{Bi}dt\;\;\;(i=x\;\mathrm{or}\;y)$$

The calculated magnetic flux density is the average value of the magnetic flux density in the area where the probe is located. Because the spacing between a pair of probes is only 9 mm, which is relatively small compared to the size of the iron core to be measured, the magnetic characteristics measured by the probe can be approximated as the magnetic characteristics of the center position of the probe. 

In addition, the sensor includes a 5 mm square Hall element, 2SA-10, Sentron, which is close to the surface of the core to achieve the induced emf *e*H across the Hall element in the air during the measurement. According to Faraday’s law and the boundary condition on the interface, ***B*** and ***H*** signals can be computed by

(11) $$\begin{array}{ll} B_{x,\;y} & =\;\frac{2}{s}\int_{0}^{T}e_{B_{x,\;y}}dt\\ H_{x,\;y} & =\;k_{i}\int_{0}^{T}e_{H_{x,\;y}}dt \end{array}$$

where *s* is the effective area turns, which is the product of the material thickness and the distance between two pins; *k*_i_ is the coefficient of the Hall element; and *T* is the period of the exciting voltage. 

**Figure 10:**
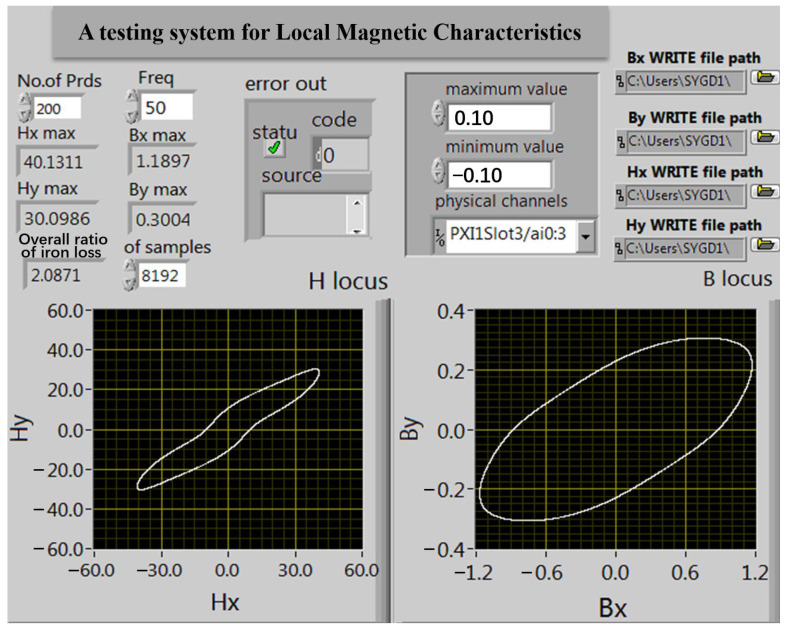
Interface of detection system of local magnetic property.

**Figure 11:**
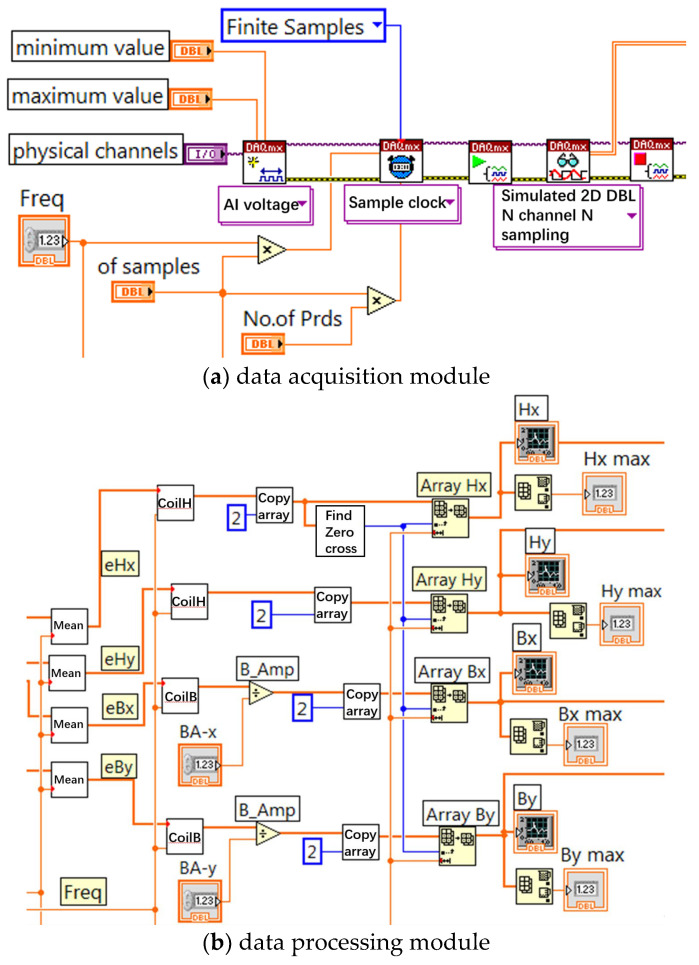

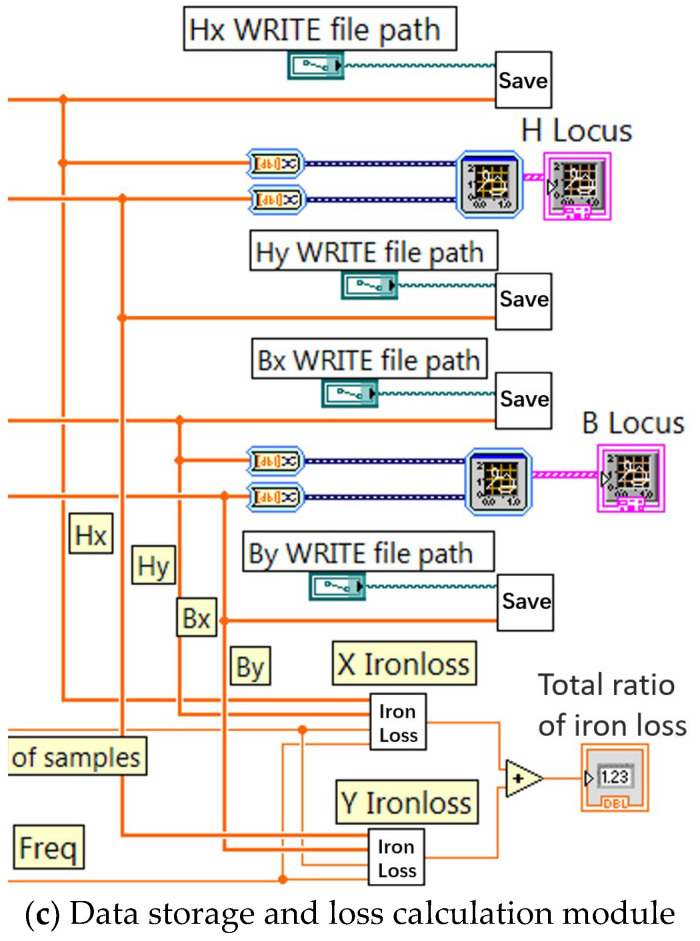
Program block diagram of LabVIEW.

The measurement system adopts virtual instrument technology and LabVIEW programming in software design. The front panel of the program written through LabVIEW is shown in Figure 10. The program diagram of LabVIEW software is composed of three parts. As shown in Figure 11, the details are as follows:

Data acquisition section: In this part, the DAQmx Create Virtual CHannel analog voltage input channel of the acquisition card and four virtual channel paths collect *e_Hx_*, *e_Hy_*, *e_Bx_*, and *e_By_* signals. First, the maximum voltage allowed through is set to ±10 V. Then, the DAQmx Timing module is called to select the limited sampling mode with the frequency set at 50 Hz and the sampling number at 8192. The DAQmx Start Task module program is called to start data collection. Next, the DAQmx Read module is called to read the data collected in the four channels. Finally, the DAQmx Clear Task module is called to clear the task. This part also adds the program error prevention function, when the program running error will be timely displayed and will stop the program running.

Magnetic field strength and flux density signal calculation part: First, the collected signals are split through the index array; the four signals are digitized; and then *H_x_, H_y_*, *B_x_*, and *B_y_* signals are obtained through the magnetic field intensity and flux densitometer operator program; then, the waveform curves of the four signals are drawn through the waveform chart. In this part, the peak *H_x-max_*, *H_y-max_*, *B_x-max_*, and *B_y-max_* values of the signal are obtained through the index of the maximum value.

Data storage and loss calculation module part: Based on Formula (12), the obtained *H_x_*, *B_x_* and *H_y_*, *B_y_* signals are obtained through the specific loss calculation subroutine to obtain the specific loss values in the *x* and *y* directions, and finally, the total specific loss values are added. At the same time, in order to facilitate the post-processing of measurement data, a data storage module is added to this part, which stores magnetic field intensity signals *H_x_*, *H_y_* and magnetic flux density signals *B_x_*, *B_y_* after the program is finished.

(12) $$\begin{array}{ll} P & =\frac{1}{\rho T}\int_{0}^{T}H\frac{dB}{dt}\\ & =\frac{1}{\rho T}\int_{0}^{T}\left(H_{x}\frac{dB_{x}}{dt}+H_{y}\frac{dB_{y}}{dt}\right)dt\;\;(W/\mathrm{Kg}) \end{array}$$

**Figure 12:**
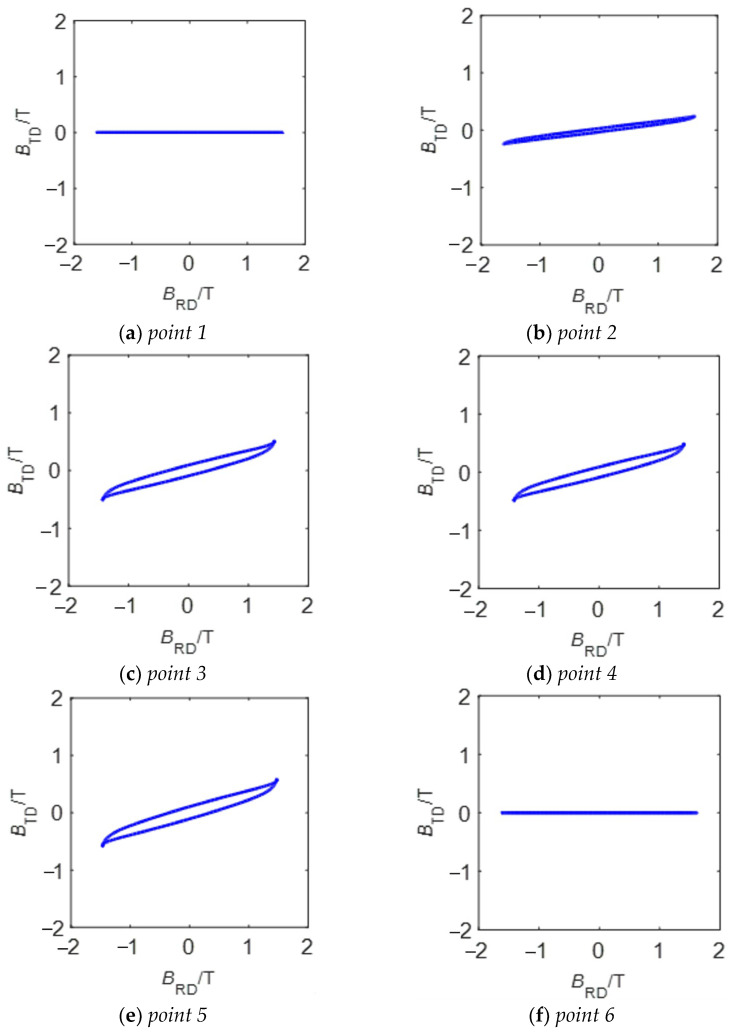
The measured results of localized magnetic characteristics at points of interest for a nominal induction *B*_NOM_ = 1.7 T.

Figure 12 shows the measured ***B*** trajectories at “*point 1–6*”. The alternating magnetizations are presented at “*point 1*” and “*point 6*” in the yoke and limb, while the rotational magnetization mainly occurs at “*point 3–5*” of the corner.

## 4. Conclusions

In this paper, a test method for magnetostrictive properties of the laminated core considering DC bias is proposed, which solves the problem of analyzing local strain characteristics of the actual core. A two-limb, one-phase transformer core with a multistep-lap construction of four steps is built to measure the localized magnetostriction. The position of clamps has great influence on the magnetostriction of the core. When the clamp is fixed in the center of the yoke, the minimum magnetostriction is achieved. The existence of DC bias increases the magnetostriction of the core, the contractive strain is produced in the RD of core and the elongated strain is produced in the TD, the peak-to-peak value of the contractive strain is larger than that of the elongation, and the strain in the corner region is greater than those in the limb and yoke. Rotating magnetization is found in the corner region by the self-developed ***B***-***H*** vector sensor, which is one of the reasons for the large magnetostriction in the corner region. The study of magnetostrictive characteristics of the laminated core can help to calculate the vibration deformation of electrical equipment such as transformers and motors more accurately, and provide a reference for the study of vibration and noise reduction of electrical equipment.

## Figures and Tables

**Table 1:** Device parameters of magnetostrictive testing system.

Device Name and Type	Performance Parameter	Parameter Values
Triaxial strain gauge KFG-5-120-D17-11	sensitivity coefficient	2.10 ± 1.0%
	Sensitive grid length	10 mm
	Sensitive gate width	3 mm
	Per-axis resistance	120.4 ± 0.4 Ω
	Base diameter	21 mm
	operating temperature range	−196~150 °C
Strain bridge box DB-120A	Bridge arm resistance	120 Ω
strain amplifier DPM-911B	magnification	100 times

**Table 2:** Comparison of contraction principal strain *λ*_−pp_ at six points of interest when different clamping positions are applied on the single-phase core magnetized at 1.5 T.

Clamping Position	*Point 1*	*Point 2*	*Point 3*	*Point 4*	*Point 5*	*Point 6*
A	1.41	1.42	2.39	2.85	2.28	1.48
B	1.43	1.65	2.25	2.76	2.08	1.45
C	1.46	1.71	2.41	2.92	2.16	1.39

**Table 3:** Comparison of peak-to-peak values of principal strain between single sheet and column region of laminated core under different magnetization conditions (μm/m).

*I*_DC_ = 0 A	1.4 T	1.5 T	1.6 T	1.7 T
single sheet	1.11	1.27	1.47	1.54
laminated core	1.22	1.43	1.72	2.19
amplification	9.9%	12.5%	17.0%	42.2%
***I*** **_DC_ = 0.1 A**	**1.4 T**	**1.5 T**	**1.6 T**	**1.7 T**
single sheet	1.40	1.57	1.72	1.77
laminated core	1.54	1.76	1.98	2.44
amplification	10.1%	12.1%	15.1%	38%
***I*** **_DC_ = 0.2 A**	**1.4 T**	**1.5 T**	**1.6 T**	**1.7 T**
single sheet	1.58	1.72	1.80	1.84
laminated core	1.76	1.94	2.32	2.576
amplification	11.3%	12.8%	28.8%	40%

## Data Availability

Not applicable.
